# LoRa-Based IoT Network Assessment in Rural and Urban Scenarios

**DOI:** 10.3390/s23031695

**Published:** 2023-02-03

**Authors:** Aikaterini I. Griva, Achilles D. Boursianis, Shaohua Wan, Panagiotis Sarigiannidis, Konstantinos E. Psannis, George Karagiannidis, Sotirios K. Goudos

**Affiliations:** 1 ELEDIA@AUTH, School of Physics, Aristotle University of Thessaloniki, 54124 Thessaloniki, Greece; 2 Shenzhen Institute for Advanced Study, University of Electronic Science and Technology of China, Shenzhen 518110, China; 3 Department of Electrical and Computer Engineering, University of Western Macedonia, 50131 Kozani, Greece; 4 Department of Applied Informatics, School of Information Sciences, University of Macedonia, 54636 Thessaloniki, Greece; 5 School of Electrical and Computer Engineering, Aristotle University of Thessaloniki, 54124 Thessaloniki, Greece

**Keywords:** Internet of Things (IoT), long-range network, smart agriculture, smart city, low-power wide-area network (LPWAN), data extraction rate (DER), network energy consumption (NEC)

## Abstract

The implementation of smart networks has made great progress due to the development of the Internet of Things (IoT). LoRa is one of the most prominent technologies in the Internet of Things industry, primarily due to its ability to achieve long-distance transmission while consuming less power. In this work, we modeled different environments and assessed the performances of networks by observing the effects of various factors and network parameters. The path loss model, the deployment area size, the transmission power, the spreading factor, the number of nodes and gateways, and the antenna gain have a significant effect on the main performance metrics such as the energy consumption and the data extraction rate of a LoRa network. In order to examine these parameters, we performed simulations in OMNeT++ using the open source framework FLoRa. The scenarios which were investigated in this work include the simulation of rural and urban environments and a parking area model. The results indicate that the optimization of the key parameters could have a huge impact on the deployment of smart networks.

## 1. Introduction

In recent years, Internet of Things (IoT) technologies and techniques have been developed to cope with modern requirements. Smart cities [1], smart homes and buildings [2], healthcare [3], manufacturing [4], and smart agriculture [5] are some of the most notable areas where IoT technologies are being adopted to address many challenges and improve the way we live. Everyday life devices are being equipped with sensors that communicate through the Internet. Based on Cisco, it is predicted that there will be 500 billion Internet-connected devices by 2030 [6].

The key requirement for data access between devices is to cover large distances and consume less power. The low-power wide-area network (LPWAN) architecture is one of the most prominent in that field, and long-range (LoRa) technology supported by the low-power wide-area networking (LoRaWAN) specification has already been adopted in numerous systems in wireless communication. A LoRa network consists of two distinct parts: LoRa and LoRaWAN. Each component corresponds to a different layer of the protocol stack.

LoRa’s design favors long-range applications that require a low rate of transmission and low energy consumption [7]. LoRa operates in the industrial, scientific, and medical (ISM) bands (from 863 to 870 MHz in Europe, from 902 to 928 MHz in the USA, and from 470 to 510 MHz in Asia).

LoRaWAN is a communication protocol [8] that uses an ALOHA-based MAC protocol. That way, the LoRa end-devices comply with the practical requirement for low complexity. LoRa end-node devices and gateways communicate over the physical layer, but there is no association between nodes and a specific gateway. Data from end nodes can be received by any gateway within a definite communication range, and the messages are forwarded toward the network server using the Internet protocol. Finally, the messages are delivered to the application server.

In our preliminary work [9], we simulated an open-field cultivation scenario using FLoRa and evaluated the performance of the network. The motivation for our work stems from the above-mentioned discussion and from the fact that we want to thoroughly investigate the most important parameters that affect the operation of LoRa networks in various simulations using the common path loss models in different environments. As such, we can evaluate the performance of LoRa networks under different conditions and draw a generalized conclusion about the efficiency of the network in urban and rural environments.

In this work, we focus on the assessment of performance metrics in LoRa networks under different propagation and environmental scenarios. We selected the two most common path loss models that are used to simulate various LoRa networks in the rural environment, and we explored how the selection of the technical parameters affects the performance of each scenario network. Moreover, we decided to extend the research to an urban area by modeling a wide variety of nodes. We also used the Oulu path loss model to compare different propagation scenarios. Finally, we modeled a dense network of nodes with the same technical characteristics to simulate a smart parking area that can be found in every modern city, and we compared the performance of this network to the previous ones.

The remainder of the paper is structured as follows. Section 2 presents the work directly related to this paper. The problem definition and the simulation scheme are provided in Section 3. In Section 4, the authors analyzed the simulation scenarios and evaluated the results. Finally, the conclusions of this study are summarized in Section 5.

## 2. Related Work

According to the relevant literature, there have been various studies on LoRa networks. Some of these have focused on LoRa network scalability. In [10], the authors released the LoRaSim simulator to study the scalability and performance of LoRa networks through simulation. They developed models that describe LoRa communication behavior, and they determined that LoRa networks can scale well by adding more gateways and/or by selecting dynamic transmission parameters. This was further extended in [11], in which the authors presented LoRaWANSim, a tool that employs bidirectional communication. Georgiou and Raza in [12] proposed a stochastic geometry framework to evaluate the behavior of a single gateway LoRa network. They found that, with an increasing number of end devices, the coverage probability decreased exponentially due to interfering signals that used the same spreading factor. In a similar study, the authors in [13] highlighted that the network scalability is more precise under the combined impact of co-SF and inter-SF interference. In [14], in the case of a single-gateway LoRaWAN deployment, the scalability was examined in terms of the number of nodes per gateway. The authors developed a simulation model that measures the impact of interference to determine the scalability of a single gateway. Furthermore, the authors in [15] used the ns-3 module to analyze the scalability. The results show that the allocation of the network parameters to nodes has a huge impact on the performance of LoRaWAN networks. Moreover, this work examines the capacity for various traffic types. In [16], the scalability of the network increases by using a new medium access protocol in a MATLAB simulator. In [17], a literature overview was presented, and various performance determinants were analyzed on LoRa-based networks.

Various experimental tests have been performed in real-world environments. LoRa networks have been studied in cases ranging from indoor [18,19] and urban/suburban [20,21] scenarios to rural [22] and mountain [23,24] environments. In [25], the researchers provided a comprehensive evaluation of LoRa networks in urban, suburban, and rural environments, considering both static and dynamic conditions. The authors in [26] evaluated the coverage and simulated the path loss model in urban, forest, and coastal environments. In [27], a path loss model based on experimental scenarios was proposed to assess the efficiency of LoRa networks in urban and rural environments in terms of coverage. The work presented in [28] focused on the evaluation of the transmission performance and the link quality of a network considering the deployment scenario and the parameter configuration. In [29], a smart building scenario was implemented to appraise the communication performance of LoRa networks without considering the power consumption. The authors in [30] presented a theoretical study and an experimental evaluation of a LoRa network. Moreover, the impact of the coding rate of the communication link is discussed in this work. In [31], the authors developed the FLoRa simulation tool to implement and evaluate the adaptive data rate (ADR) mechanism in LoRa networks. In [32], the authors evaluated the impact of SF on performance. Another method to improve the performance based on SF network clustering was presented in [33], and the authors in [34] developed an algorithm to further improve the efficiency of the LoRa network compared to the ADR algorithm.

## 3. Problem Definition

### 3.1. Evaluation Metrics

Based on the related work that was presented, we decided to assess the effectiveness of LoRa networks using two evaluation metrics:

- Data extraction rate (DER) is defined as the amount of messages which were received correctly divided by the number of messages that were sent to the server. DER is computed between 0 and 1. When the ratio is closer to 1, it means that the LoRa network is working more efficiently.
- Network energy consumption (NEC) is defined as the energy consumed by the network divided by the number of successfully received messages. A low value for NEC implies a more efficient network.

### 3.2. Path Loss Models

To model radio wave propagation, most researchers are using the log-normal shadowing model, which depends on empirical data [10]. The Oulu city model [18] and the Okumura–Hata (OH) model have been used to assess the coverage in LoRa networks [25,35]. In our research, we chose to look more closely at these three different path-loss models. A short summary of each model is given.

#### 3.2.1. Log-Distance Path Loss Model with Shadowing

The log-distance path loss model with shadowing is widely used in wireless communication to model the obstruction on the propagation path between a base station (BS) and a mobile station (MS). The equation is given as follows:

(1) $$PL\left(d\right)=\bar{PL}\left(d_{0}\right)+10\times n\times log\frac{d}{d_{0}}+X_{\sigma }$$

where the PL(d) stands for the path loss, $PL(d_{0})$ is the mean path loss measured in dB, *n* is used for the path loss exponent, $X_{s}$ is the loss due to shadow fading with a zero-mean Gaussian distribution, and σ denotes the standard deviation. In this work, *n* was set to 2 and sigma was set to 5 dB in order to simulate the rural environment, whilst *n* was selected equal to 2.08 and σ was set to 3.57 dB to simulate the urban environment.

#### 3.2.2. The Oulu Path Loss Equation

Based on experimental data, the authors in [36] provided the Oulu path loss model using the following equation:

(2) EPL=B+α×logR

where EPL is the expected path loss, *B* is the path loss in dB, α describes the path loss exponent, and *R* is the distance between the node and the base station divided by the 1 km reference distance.

The standard deviation of shadow fading describes a deviation between the measured path loss and expected path loss and is computed as follows

(3) $$\sigma_{SF}=std\left(PL-EPL\right)$$

The city of Oulu is a medium-sized city with high residential buildings in the center, located one the seashore and with a mainly flat terrain. The approach provided by this area can be used to model many similar urban environments all over the world. The measurements were conducted using a mobile node on the roof of a car moving over the ground and using a node on a boat over the water. The base station was on the roof of the University of Oulu, 24 meters above sea level.

#### 3.2.3. Okumura–Hata Path Loss Model

The Okumura–Hata empirical model is a widely used radio propagation model for predicting path loss. The model is appropriate for linking distances from 1 to 20 km and a frequency range of 150–1500 MHz. The user antenna heights range from 1 to 10 m and the BS antenna height ranges from 30 to 200 m. The Okumura–Hata model is popular for its accuracy and simplicity. This model has the advantage of being adjusted in many different environments from open areas to large cities by selecting the appropriate variant, as is apparent from the following equations.

The Okumura–Hata path loss equations are modified as follows:

(4) PL=A+B×logR+C

With regard to an urban environment, the two following Okumura–Hata variants can exist

(5) $$\begin{array}{cc} \; & \;Lurban=69.5+26.16\times logf_{c}-13.82\times logh_{b}-a\left(h_{m}\right)\\ \; & \;+(44.9-6.55\times logh_{b})\times logR \end{array}$$

For small or medium-sized cities,

(6) $$a\left(h_{m}\right)=\left(1.11\times logf_{c}-0.7\right)\times h_{m}-\left(1.56\times logf_{c}-0.8\right)$$

and for large cities,

(7) $$a\left(h_{m}\right)=\left\{\begin{array}{cc} (8.29\times {\left(log\left(1.54\times h_{m}\right)\right)}^{2}-1.1, & f_{c}\leq 300\mathrm{MHz}\\ (3.2\times {\left(log\left(11.75\times h_{m}\right)\right)}^{2}-4.97, & f_{c}>300\mathrm{MHz} \end{array}\right.$$

Under the sub-urban environment,

(8) $$L_{suburban}=L_{urban}-2\times (log(\frac{f_{C}}{28}{))}^{2}-5.4$$

Under the free/open/rural environment,

(9) $$L_{open}=L_{urban}-4.78\times {\left(logf_{c}\right)}^{2}-18.33\times logf_{c}+40.94$$

where $L_{urban}$ is the path loss in urban areas, $L_{suburban}$ is the path loss in suburban areas, and $L_{open}$ is the path loss in open-rural areas in dB. $h_{b}$ is the height of the base station antenna, and $h_{m}$ is the height of the mobile station antenna in m. Moreover, $f_{c}$ is the frequency of the transmission in MHz and *R* is the distance between the station and the mobile stations in km. Finally, the $a(h_{m})$ is the correction factor for mobile antenna height.

### 3.3. Simulation Tools

As LoRa technology is widely used in IoT applications, various simulation environments were developed to study, evaluate, and optimize LoRa networks before their implementation. A short overview of the most commonly used simulators that have been developed over the years is presented in this section.

- LoRaSim is a discrete event simulator developed to study the scalability in LoRa networks and to model collisions. This tool is written in Python and allows modeling in a 2-dimensional grid. By selecting a number of parameters such as the number of nodes and base stations, the radio settings, and the simulation time, we can have information regarding the collisions, transmissions, and total energy spent [10].
- The Framework for LoRa (FLoRa) is an open source simulation tool based on the OMNeT++ (ver. 5.3, OpenSim Ltd., Budapest, Hungary) [37] discrete event simulator and the INET framework [38]. FLoRa provides a precise model of the physical layer taking into consideration the capture effect as well as the collisions and also implements the MAC layer. Moreover, FLoRa includes the various modules of a network such as nodes, gateway(s), and server(s), supports bi-directional communication and enables end-to-end simulations. One or more gateways can be simulated in the model and can receive transmissions from nodes on multiple channels. The gateways communicate with the server over the IP protocol. The tool is written in C++ and NED language and is used to simulate LoRa networks. Moreover, it provides the statistics for energy consumption in every node considering the three states (transmitting, receiving, and sleeping) of a LoRa radio [31].
- The NS-3 module is a discrete-event network simulator written in C++ and Python. This module is used to assess the performance of a network in various cases because it can simulate many aspects of a network such as the layers of the system and the protocols that are used [15].
- LoRaWANSim is a LoRaWAN simulator that is written in MATLAB. This simulator was developed to characterize the behavior of LoRaWAN networks. In LoRaWANSim, we can model and also modify parameters related to the PHY layer and the LoRaWAN protocol [11].

**Figure 1:**
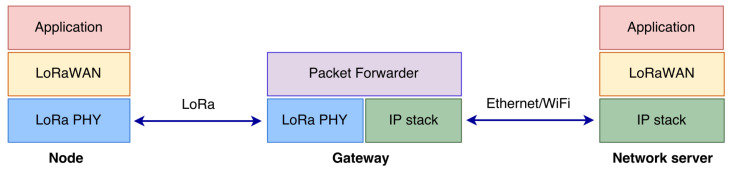
Modules in FLoRa and the corresponding protocol stack [31].

Considering the requirements of our study, the FLoRa open source simulation tool was selected. As shown in Figure 1, the tool was used to model the LoRa physical layer and the LoRaWAN MAC protocol, as well as the network elements such as network servers, nodes, and gateways. The module describing the energy consumption was also used.

## 4. Numerical Results

In this part, we analyzed the various scenarios we implemented to assess the performance of LoRa Networks. In every simulation, we assumed that the nodes were uniformly distributed on a square deployment area, whereas the gateways were arbitrarily placed. Each scenario lasted 7 days, and the physical layer of the LoRa network was simulated in each simulation environment by choosing the right European regional parameters.

Five configuration parameters were selected to determine the energy consumption, the transmission range, the data rate, and the noise durability of a LoRa transmission [10].

- Transmission power (TP). TP can be set between −4 and 20 dBm. When the TP increases, the energy consumption of the network and the signal-to-noise ratio (SNR) are also increased.
- Bandwidth (BW). BW can be set to 125 kHz, 250 kHz, or 500 kHz. A bigger BW provides a higher data rate but decreases the radio sensitivity.
- Spreading factor (SF). SF can be in the range of 7–12. A higher SF improves the communication range but increases the energy consumption.
- Carrier frequency (CF). CF can be selected between 137 MHz and 1020 MHz according to the ISM band in the region of operation.
- Coding rate (CR). CR can be configured to 4/5, 4/6, 4/7, or 4/8 to provide security from interference. By choosing a higher CR, the network becomes more reliable, but the air time increases.

### 4.1. Case Study 1—Rural Environment

The rural environment was simulated by using the log-distance path loss model and the Okumura–Hata path loss model. In each scenario, the transmission power was set to 10 dBm, the bandwidth was fixed at 125 kHz, the code rate was selected as 4/8, the chosen spreading factor was 7, and the carrier frequency was selected as 868 MHz (Table 1).

#### 4.1.1. Log-Distance Path Loss Model with Shadowing

**Figure 2:**
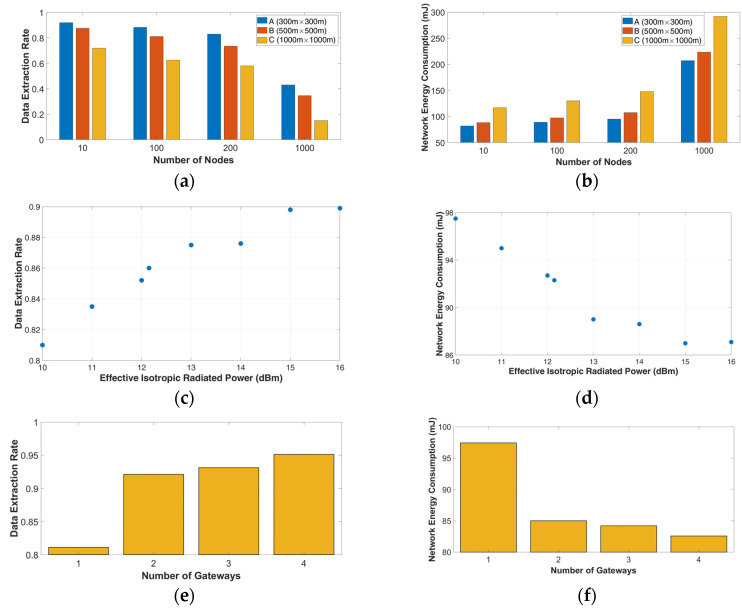
Log-normal shadowing model in rural environment. (**a**) DER as a function of the number of nodes; (**b**) NEC as a function of the number of nodes; (**c**) DER as a function of the EIRP; (**d**) NEC as a function of the EIRP; (**e**) DER as a function of the number of gateways; and (**f**) NEC as a function of the number of gateways.

*Scenario 1.1* In the first scenario, we assess the effectiveness of a LoRa network in three squared areas. The first deployment area was set to 300 m × 300 m, the second deployment area was set to 500 m × 500 m, and the third area was set to 1000 m × 1000 m. The number of nodes was increased from 10 to 1000 in each area. Figure 2a,b show that, with an increasing number of nodes, there is a decrease in the network performance, due to the more frequent incidents of collisions between the packets that were sent from nodes with identical characteristics. As we can obtain by increasing the nodes count from 10 to 1000 in the deployment area B, the collisions are increased by 64%. Moreover, the results of the simulations indicate that the performance metrics (DER, NEC) we obtained are affected by the dimensions of the deployment area. This occurs because, as the dimension of the deployment space is increased, the exact number of nodes must be spread across a larger space.

*Scenario 1.2* In the second scenario, the impact of the effective isotropic radiated power was explored. We modeled 100 uniformly distributed nodes in a 500 m × 500 m deployment area, and a single gateway was located at the point (x = 0, y = 0). We simulated various antennas, such as the isotropic antenna, the 0.173 m dipole antenna, and isotropic antennas with constant gain from 1 dBi to 6 dBi. Figure 2c,d show that the obtained performance metrics of the network are improved as the EIRP increases. Moreover, we can detect an increase of 4.41% by replacing the antenna from dipole (EIRP = 12.15 dBm) to isotropic antenna with a constant of gain 6 dBi (EIRP = 16 dBm).

*Scenario 1.3* In this series of simulations, we appraise the performance of 100 LoRa nodes deployed in the same 500 m × 500 m area using multiple gateways. Firstly, a single GW_0_ was located at the point (x = 0, y = 0). Then, a second GW_1_ was placed at (x = x_max_/2, y = y_max_/2). In the next set of simulations, we used the first GW_0_, a GW_2_ located at (x = x_max_/2, 0), and a GW_3_ located at (0, y = y_max_/2). For the last set, we used all of the previous gateways in the deployment area. This gateway layout was selected for simplicity. The performance of the network is increased by using more gateways, as shown in Figure 2e,f. When the number of gateways is increased from 1 to 2, we obtained an increase of 12.75% in the network performance. This increase is affected by the location of the second gateway. We selected to place it at the point (x = x_max_/2, y = y_max_/2) to reduce the distance between the gateways and the nodes. The simulations show that, if we place the second gateway at the other side of the deployment area, for example, at the point (x = x_max_, y = y_max_), the increase in the performance is changed to 11.2%.

#### 4.1.2. Okumura–Hata Path Loss Model

**Figure 3:**
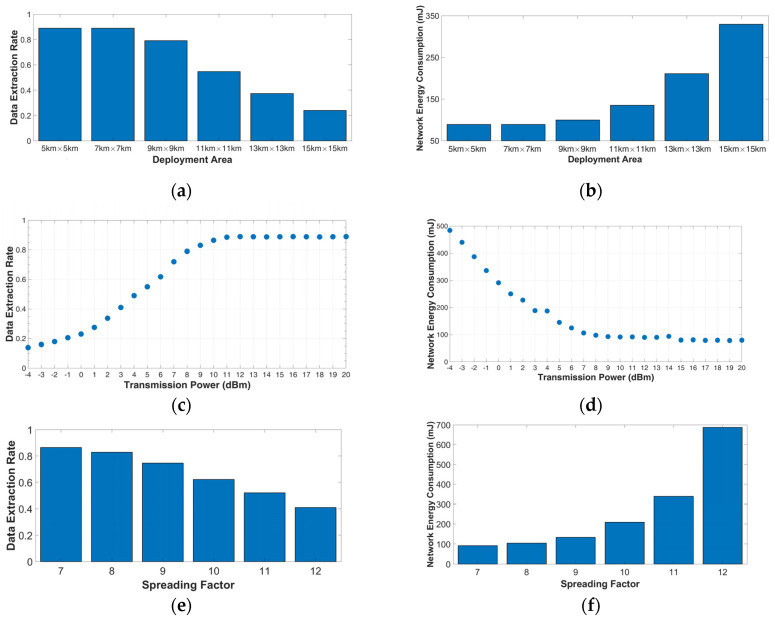
Okumura–Hata model in the rural environment. (**a**) DER as a function of the size of the deployment area; (**b**) NEC as a function of the size of the deployment area; (**c**) DER relative to the transmission power; (**d**) NEC relative to the transmission power; (**e**) DER relative to the spreading factor; and (**f**) NEC relative to the spreading factor.

**Figure 4:**
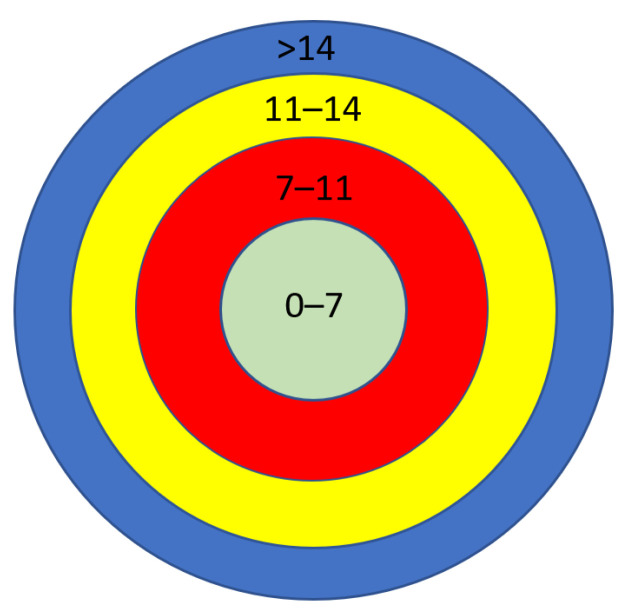
The area divided into zones.

*Scenario 1.4* In the next set of simulations, six different deployment areas were selected. The smallest deployment area was set to 5 km × 5 km, the largest deployment area was set to 15 km × 15 km, and the rest of the deployment areas were selected between those boundaries. A gateway was located at (x = 0, y = 0) and 100 nodes were placed at each deployment area. Figure 3a,b show that the efficiency of the network decreases by expanding the deployment area’s size. Regarding the percentage of the packets that arrive at the gateway having a power level below the minimum sensitivity level, we can divide the area into four zones, as shown in Figure 4, namely 0–7 km, 7–11 km, 11–14 km, and more than 14 km. Within a 7 km range from the gateway, every node can successfully communicate with the gateway. In the 7–11 km range, 2.89% of the nodes cannot reach the gateway. In the third zone, 11–14 km, the figure rises from 2.89% to 24%. Finally, above 14 km, the number of nodes that cannot communicate with the gateway has significantly increased.

*Scenario 1.5* In this scenario, we analyze the influence of the TP on the main performance metrics of a LoRa network. We placed a gateway at (x = 0, y = 0) and 100 nodes uniformly distributed at the deployment space that was selected to be 8 km × 8 km. Figure 3c,d show that with a growing value from -4 dBm to 10 dBm, the performance of the network increases. For example, by increasing the TP from 2 dBm to 6 dBm, the performance is increased by 45.3%. A threshold is obtained at 11 dBm and that means that we can achieve the maximum performance of the network in the specific deployment area that we have simulated, by choosing between a big range of transmission power values.

*Scenario 1.6* In this scenario, we selected the same layout as in Scenario 1.6. The TP was set to 10 dBm and we obtained the performance of the network by changing the value of SF. Figure 3e,f show that, while we increase the value of the SF, the time on air is also increased. Consequently, we observe an increase in the energy consumption of the network. For example, by changing the SF from SF11 to SF12, the energy consumption increases by 50.76%.

### 4.2. Case Study 2—Urban Environment

The urban environment was simulated using the log-distance path loss model, the Okumura–Hata model, and the Oulu city model. To simulate the urban environment, the transmission power was chosen between 2 and 14 dBm, the code rate was set to 4/8, the bandwidth was set to 125 kHz, the carrier frequency was selected to 868 MHz, and the spreading factor was in the range of 7–12 (Table 2).

#### 4.2.1. Log-Distance Path Loss Model with Shadowing

**Figure 5:**
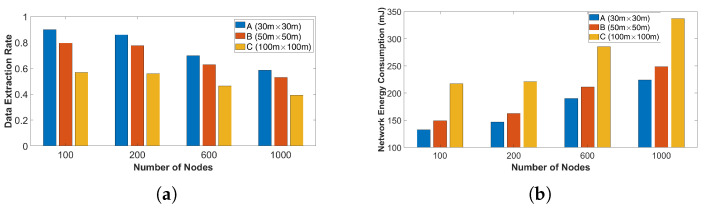
Log-normal shadowing model in the urban environment: (**a**) DER as a function of the number of nodes; and (**b**) NEC as a function of the number of nodes.

*Scenario 2.1* In this set of simulations, three different deployment areas were selected. Deployment area A was set to 30 m × 30 m, B was set to 50 m × 50 m, and C was set to 100 m × 100 m. The performance of a LoRa network was evaluated for an increasing number of nodes. Figure 5a,b show that, while the number of nodes increases from 100 to 1000, the efficiency of the network is decreasing and comes as a result of the increase in the number of collisions between the packets. The number of this decrease compared to the rural environment is lower due to the range of the TP and SF values which were used to simulate the nodes in an urban environment. In the urban environment, the number of undetectable packages depends on the combination of the location, the TP, and the SF of the nodes. For example, by simulating 100 nodes in deployment area C, the percentage of the packages that reach the gateway with a power level lower than −100 dBm was almost 39%. With an increasing number of nodes, the percentage of undetectable packages was slightly reduced. As reflected by the previous simulations, the size of the deployment space affects the achieved performance.

#### 4.2.2. Okumura–Hata Path Loss Model

**Figure 6:**
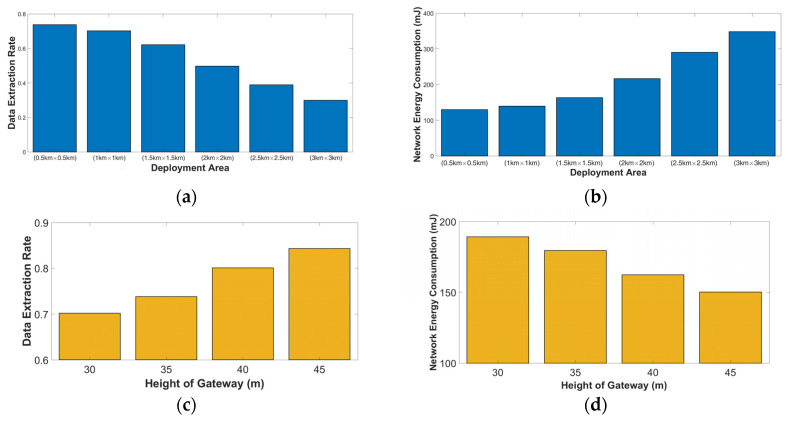
Okumura–Hata path loss model in an urban environment: (**a**) DER as a function of the size of the deployment area; (**b**) NEC as a function of the size of the deployment area; (**c**) DER relative to the height of gateway; and (**d**) NEC relative to the height of gateway.

*Scenario 2.2* Six deployment areas were modeled to evaluate the performance of the network in this set of simulations. The smallest deployment area was set to 500 m × 500 m, and the largest deployment area was set to 3 km × 3 km. A gateway was modeled at a height of 30 m from the ground at (x = 0, y = 0), and 600 nodes were located uniformly at each deployment area at a height of 1 m. Figure 6a,b show that the performance of the network slowly decreases by increasing the deployment area’s dimensions.

*Scenario 2.3* In this scenario, we changed the height of the gateway in deployment area B. When we raise the height of the gateway, the performance of the network is increased, as shown in Figure 6c,d. For example, by changing the height from 30 m to 35 m, the performance is increased by 5% which is very important because we can deploy the same network in a wider area (1.5 km × 1.5 km) without changing any configuration parameters.

#### 4.2.3. Oulu Path Loss Model

**Figure 7:**
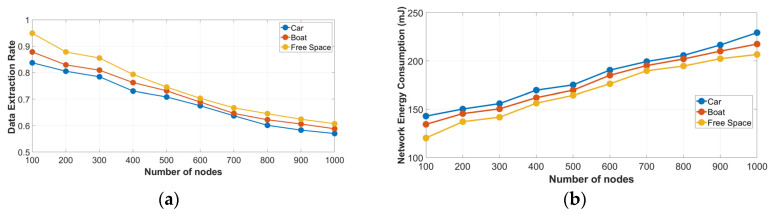
Oulu city path loss model in an urban environment: (**a**) DER as a function of the number of nodes; and (**b**) NEC as a function of the number of nodes.

*Scenario 2.4* At this setup, we used the Oulu city path loss model to evaluate the performance of a LoRa network (Table 3). The deployment area was set to be 2 km × 2 km and a gateway was located at the point (x = 0, y = 0) at 24 m from sea level. Figure 7a,b show the results for a growing number of nodes by simulating the different propagation models.

The performance of the network decreases when we increase the number of nodes due to the frequent incidents of collisions between the packets. As expected, the best results are obtained using the free space path loss model. On the other hand, when the car model was used, we detected the lowest value in the performance of the network. The car model was selected to simulate an area where buildings and other physical obstacles are blocking the path between the nodes and the gateway.

### 4.3. Case Study 3—Parking Model Environment

In the last series of simulations, we attempted to evaluate the performance of a parking model environment. The deployment area was set to 100 m × 100 m in every simulation set. Firstly, to model 600 nodes in the urban environment, we used the European regional parameters. Then, for the same number of nodes, we selected the TP to be fixed at 10 dBm and the SF at 7 to simulate a parking area with a large number of identical nodes. Finally, 50 nodes were simulated using the appropriate parameters to model the LoRa physical layer in the rural environment.

**Figure 8:**
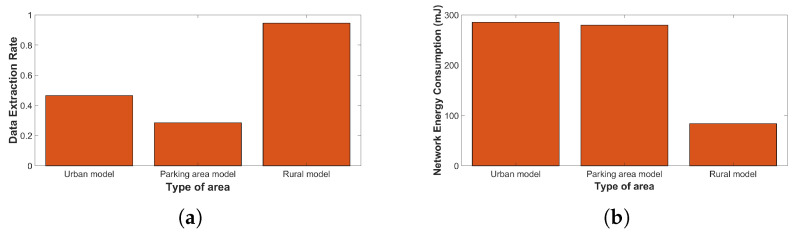
Parking model: (**a**) DER relative to the type of area; and (**b**) NEC relative to the type of area.

Figure 8a,b show the results for the three different cases. Comparing the urban model to the parking area model, we can see that the data extraction rate of the network is decreased by 38.79% when we select identical nodes. This decrease is caused due to a large number of collisions between the packets. Regarding the energy consumption of the network, we observe that it slightly decreased by 2% in the parking area model. Finally, we can obtain that the performance in the rural environment is significantly high and the data extraction rate is very close to the highest value.

## 5. Conclusions

In this work, an assessment of LoRa networks was presented in three different use cases: the rural environment, the urban environment, and the parking model environment. Firstly, we explored the way in which the network density impacts performance in LoRa networks in both rural and urban environments. The results show that, while the amount of nodes increases, the network’s performance decreases. This is more obvious in the implementation of rural environments because we modeled nodes with identical technical characteristics. Moreover, we explored the impact of EIRP in our model and highlighted the importance of the number and the location of the gateways in the performance of LoRa networks by selecting the log-distance path loss model with shadowing. The impact of the transmission power and the spreading factor was explored using the Okumura–Hata path loss model. By increasing the transmission power, the number of the delivered packages was also increased until the stimulation upper threshold of 11 dBm was reached. While we increased the spreading factor from SF 11 to SF 12, the energy consumption of the network was increased by 50.76%. Furthermore, the results show that the selection of the dimensions of the deployment area and the height of the gateway are very important to implement more efficient networks. By choosing the Oulu path loss model, we managed to record the impact of the propagation model on the network performance. Finally, we simulated a parking model area to underline the network behavior by changing the environmental parameters of the modeled area. As future work, the current project can be extended in several directions, such as performing experimental evaluations in a real-world environment to assess the simulation results. To develop a full picture of the behavior of a LoRa network in specific environments, for example, in a smart agriculture scenario or/and in an urban area in Greece, we are planning to simulate and experimentally implement LoRa networks in order to define the numeric parameters of the path loss model taking into consideration the different type of crops in the agriculture scenario and the specifications of the urban area, respectively. Finally, further research should be undertaken to investigate the improvement of the performance of LoRa networks using optimization algorithms.

## Figures and Tables

**Table 1:** Simulation parameters in the rural environment.

Parameter	Value
Transmission power (TP)	10 dBm
Carrier frequency (CF)	868 MHz
Spreading factor (SF)	7
Code rate (CR)	4/8
Bandwidth (BW)	125 kHz

**Table 2:** Simulation parameters in urban environment.

Parameter	Value
Transmission power (TP)	2–14 dBm
Carrier frequency (CF)	868 MHz
Spreading factor (SF)	7–12
Code rate (CR)	4/8
Bandwidth (BW)	125 kHz

**Table 3:** Simulation parameters in the city of Oulu [36].

Metric	Car	Boat	Free Space
Path loss exponent (n)	2.32	1.76	2.00
Path loss intercept (B)	128.95	126.43	91.22
Shadow fading ($\sigma_{sf}$)	7.8 dB	8.0 dB	-

## Data Availability

Data sharing not applicable.
